# Some Problems in Surgical Treatment of Abdominal Conditions

**Published:** 1921-06

**Authors:** Forbes Fraser

**Affiliations:** Surgeon, Royal United Hospital, Bath, and formerly Consulting Surgeon, B.E.F.


537435

The Bristol

Medico-Chirurgical Journal.

" Scire est nescire, nisi id me
Scire alius sciret."

MARCH AND JUNE, 1921.

### SOME PROBLEMS IN SURGICAL TREATMENT OF ABDOMINAL CONDITIONS.

BY

FORBES FRASER, C.B.E., F.R.C.S.,

*Surgeon, Royal United Hospital, Bath, and formerly Consulting Surgeon, B.E.F.*

---

At the present day there exist many interesting problems concerning the surgical treatment of abdominal conditions. The correct method of operation for chronic ulcers of the stomach, the treatment of intestinal stasis, of perforation of gastric and duodenal ulcers, of peritonitis, and the vexed question of peritoneal drainage are all matters which might lead to interesting discussion. The present remarks are concerned only with those cases of chronic disorders of the digestive apparatus which come to the surgeon on account of the failure to give relief of prolonged medical treatment and dieting. Such cases may conveniently be grouped together under the term " Chronic Abdomen."

For years past many of the best brains of our profession have been working at the symptomatology of the various conditions which may be included under the above term, and the value of such work as that of Moynihan and of Hurst on duodenal and gastric ulcers, gall-bladder conditions and appendix dyspepsia cannot be over-estimated.

The classical chain of symptoms associated in our minds with duodenal ulcer—the occurrence of pain at a definite interval of two to three hours after food, the relief of pain by a meal, the regular recurrence of pain in the small hours of the morning, and so on—leads to a correct diagnosis in the larger proportion of cases.

Nevertheless, in spite of all the brilliant work of these and many other observers, it all too frequently happens that the diagnosis made after most careful questioning of the patient and most thorough physical examination is proved to be wrong at the subsequent operation, which may reveal a condition of affairs almost entirely unsuspected.

To quote a few cases illustrating this uncertainty :—

1. An elderly man, thin and emaciated, was admitted to hospital having for two years complained of epigastric pain, constantly present, always worse immediately after food, and of frequent and copious vomiting. X-ray examination showed a rather dilated, decidedly hypotonic stomach, not completely emptied ten hours after the opaque meal. At the operation there was found a large active ulcer of the first part of the duodenum and a gall-bladder full of stones. There was no organic obstruction of the pylorus. Infolding of the ulcer, gastro-enterostomy and removal of the gall-bladder gave him complete relief.

In this case an adherent appendix obviously the seat of old disease was left alone owing to the patient's condition at the operation.

2. A middle-aged woman was admitted to hospital having complained for ten months of pain coming on two hours after her meals and waking her up at one o'clock in the morning. The pain was relieved by food. Shortly before admission to hospital she had vomited some blood, and had definite melæna which lasted for a fortnight. X-ray examination showed marked hypertonicity of the stomach. At the operation no ulcer was found either in the duodenum or stomach. There was no redness of the pylorus or enlargement of glands along the greater curvature. The appendix showed no definite signs of disease, the gall-bladder was normal, but the cæcum and the ascending colon were provided in their whole length with a long mesentery, which enabled them to be completely lifted from the wound and laid on the towels on the left side of the abdomen. Fixation of the proximal colon entirely relieved her symptoms. The appendix, though not obviously diseased, was removed in case the colopexy should involve it in adhesions or cause it to be kinked.

3. A man in excellent health, who had been lying in bed for the previous month on account of a broken leg following a hunting accident, was suddenly seized with symptoms indicative of a perforated duodenal ulcer. With the exception of slight indigestion for two days previous to this catastrophe he had had no symptoms pointing to digestive disorder. At the operation a large ulcer in the first part of the duodenum was found, which had perforated ten hours previously.

In the first case, then, a chronic duodenal ulcer was present when the symptoms pointed to some entirely different condition.

In the second case no ulcer was present, though the symptoms were such as might lead one to look with confidence for such a lesion.

In the third case a large ulcer was present which must in all probability have been in existence for a considerable time without producing symptoms of any kind whatever.

Such variations from the typical clinical history of Moynihan and the typical radiological appearances of Hurst must be quite common in the experience of every operating surgeon, and their frequency leads to the inquiry whether after all an accurate symptomatology for any one organ of the several which appear to be so intimately related together can be expected to exist as a universal rule. It is interesting in this connection to compare the frequency and confidence with which gastric ulcer was formerly diagnosed in the past with the most recent view founded on the unrivalled experience of Sir Berkeley Moynihan, who now holds that gastric ulcer cannot be diagnosed by clinical signs or symptoms at all, and indeed that the only way to be sure of the presence of this condition is to see the ulcer, either at the operation or on an X-ray plate. It has long been recognised that there exists a correlation of symptoms between the stomach, the duodenum, the gall-bladder and the appendix, and that in a given condition the fault may be in one or all of these structures. So much indeed has the interchangeability of the subjective symptoms of disease of these organs become a matter of faith with surgeons, that it has often been the custom on finding the first three normal at the operation to proceed to remove a more or less normal appendix in the pious hope that this may give the patient relief. The undoubted existence of the real and well-marked condition known as appendix dyspepsia, and first described and elaborated by Moynihan, can hardly be regarded as justifying such a procedure. As pointed out by Moynihan, true appendix dyspepsia is associated with pyloric spasm, with injection of vessels at the pyloric end of the stomach, and with enlargement of the glands at the pyloric end of the greater curvature.

Yet another factor exists to which attention was first called by Wilms in 1904 and recently by Waugh, by Tyrrell Gray and by Morley, *i.e.* abnormal mobility of the cæcum and ascending colon due to the presence of a persistent mesentery. Waugh argued that an ascending colon provided with a mesentery must work at a mechanical disadvantage as compared with the same structure relatively fixed to the posterior abdominal wall, and that as the result of the long-continued strain thus produced over-distension of the cæcum and sagging downwards of the latter and of the ascending colon are brought about.

On a purely mechanical theory Waugh believes that conditions such as ulceration of the duodenum, gall-stones, dropping of the right kidney, etc., are primarily caused by the dragging of a mobile and overloaded proximal colon. Tyrrell Gray has further elaborated the possible results of this condition, but has shown that far more important than the mere mechanical drag on the structures to which the proximal colon is attached by its peritoneal investment is the influence produced upon them reflexly by stimulation of the splanchnic nerves situated in the mesentery.

What, then, is the nature of the relationship which exists between these different organs—the stomach, duodenum, gall-bladder, appendix, ascending colon and cæcum—a relationship the practical importance of which appears to be that changes occurring in one or more members may cause symptoms referable to others of the group ?

The connection may be :—

(*a*) *Mechanical*, as by bands or adhesions which directly interfere with function, or, as indicated above, by the drag of a heavily-laden and sagging bowel on the other organs to which it is attached by its peritoneal investment.

(*b*) *Toxic.*—The gall-bladder and the appendix are notoriously liable to become the harbourers of virulent organisms, and the same is true of the sagging and distended proximal colon of intestinal stasis. It may well be believed that an organ so delicately susceptible as the duodenum to physiological chemical stimuli should also be readily affected by bacterial toxins.

(*c*) *Nervous or Reflex.*—The physiological investigations of Cannon and of Elliott on the mechanism of intestinal movements throw interesting light on the problems of abdominal disease.

Elliott has shown that stimulation of the splanchnic nerve causes inhibition or relaxation of the whole small intestine with the exception of the two sphincters—the pylorus and the ileo-cæcal valve—which are thrown into a condition of tonic spasm.

The splanchnic nerves are situated in the mesentery, and Tyrrell Gray suggests that distant reflex effects well known to occur in cases of inflammation or adhesions of such an organ as the appendix can only be produced when the mesentery is involved in the morbid process.

It is, of course, impossible to draw a hard-and-fast line between the mechanical, toxic, and reflex influences which one organ may exert on another. For instance, the mechanical drag of adhesions may lead to stasis in the appendix, colon, or gall-bladder, as the case may be, and thus initiate a toxic process, which itself may act directly on the muscle fibres or secretory glands or indirectly through the splanchnic nervous system. Or the inhibitory stimuli generated in the mesentery of an inflamed appendix may in the first place, by producing pyloric spasm, prevent the escape of acid gastric juice into the duodenum and thus throw out of gear the whole of the elaborate apparatus engaged in regulating the flow of pancreative juice and bile; and in the second place, by producing relaxation of the cæcum and colon, they may cause a sagging and loaded bowel to drag heavily on the peritoneal covering of the duodenum, and thus aggravate the mischief already initiated.

This experiment of Elliott's I have seen on two occasions perfectly reproduced by disease in the human body. Both instances were cases giving typical signs and symptoms of acute intestinal obstruction, such as might be produced by strangulation of the small intestine by a band; both had profuse stercoraceous vomiting. At the operation the whole small intestine was found to be distended with the exception of the ileo-cæcal valve and the lower inch or two of the ileum, which were in a condition of acute enterospasm. No mechanical obstruction could be found, and the cause of the enterospasm in each case was an acutely inflamed appendix, the removal of which promptly relieved all symptoms.

A third case of intestinal obstruction from adhesions in the neighbourhood of the ileo-cæcal valve showed relaxation of the lower two feet of the ileum with intense contraction of the whole small intestine above, thus suggesting that splanchnic stimuli need not always produce the typical effects of Elliott's experiment, but that variations of spasm and inhibition may occur in different sections. The very important bearing of intestinal spasm and relaxation on the treatment of acute peritoneal conditions, on the causation of gastric ulcers, and on the rationale of gastro-enterostomy is convincingly set forth by Tyrrell Gray.

Another fact elucidated by physiologists which, while it perhaps does not bear directly on the present question, yet appears to me of great surgical importance, is that the normal movement of the proximal colon is not that of propulsive peristalsis but antiperistalsis. The contents of this part of this bowel are being constantly and repeatedly driven against the ileo-cæcal valve, thereby producing a central reflux current in the transverse and descending colon. In fact, a similar effect is produced in the proximal colon to that which obtains in the stomach when peristaltic waves drive its contents against the closed pylorus. In face of this physiological fact, it is easy to understand the futility of ileo-sigmoidostomy for chronic constipation, and possibly some light is also thrown on the relatively excellent results obtained after hemicolectomy as compared with the disastrous effects so frequently seen after Lane's operation of complete excision of the colon.

It has been suggested that mobility of the proximal colon can be easily determined by comparison of radiograms taken with the patient lying down during full expiration and standing up during full inspiration. My own experience leads me to believe that unless an extreme degree of mobility, say five inches, is shown by the X-ray examination, the method is of little value.

It is certain that a colon with no mesentery may glide freely on its areolar attachment to the posterior abdominal wall, but if Tyrrell Gray's arguments be accepted, such mobility is in itself of little importance. In order that the far-reaching neuro-muscular effects above described may be produced, it is necessary that the splanchnic nerves in the mesentery should be dragged on or otherwise stimulated. It is impossible to be certain of the existence of a persistent mesentery except by actual vision and examination when the abdomen is opened.

The theory that duodenal and gastric ulcers are invariably caused by the dragging of an ascending colon with persistent mesentery cannot be maintained. I have operated on two cases of definite duodenal ulcer in which no mesentery was present for this part of the bowel. That this condition, on the other hand, may sometimes be concerned in the causation of duodenal ulcer is to some extent supported by the fact that I have twice treated large, well-defined duodenal ulcers entirely by fixation of the colon, together with removal of the appendix, and both these cases have been relieved of their symptoms for over twelve months. The only saddleback ulcer of the lesser curvature on which I have operated by this method was not cured.

While, therefore, it may be conceded that persistent mesentery in the ascending colon may be one of the factors concerned in the etiology of duodenal and gastric ulcers, it certainly is not the only one, and Waugh's operation of colopexy should not take the place of well-tried measures such as gastro-enterostomy and partial gastrectomy. As an adjunct to gastro-enterostomy this operation appears likely to have a useful function.

My own experience of Waugh's operation for conditions depending on mobility of the right kidney is limited to two cases, both of which had large hydronephrosis from kinking of the ureter; in both cases the kidney was heavily infected by colon bacilli. Waugh's operation was performed on these patients and the kidney was not touched. Both have been completely relieved.

It appears probable, then, that this method of treatment may prove to be useful in cases of dragging pain associated with mobile right kidney, for which nephrorrhaphy has hitherto been considered the correct treatment, and that it may eventually replace the latter method, the results of which have been so frequently uncertain and unsatisfactory.

In that large class of chronic abdominal cases presenting gastric, duodenal, or right iliac symptoms in which at the operation no organic disease is found, the fixation of a colon and cæcum freely mobile on account of the persistence of the mesentery appears on the whole to give the best results hitherto attained by surgical measures. Failures are met with, but the same may be said of any other operation in surgery. On the other hand, in the majority of cases considerable relief is gained by the patient, and in some the results may be spoken of as brilliant.

In addition to these conditions I might mention two cases of long standing and persistent diarrhœa which had resisted all kinds of medical treatment, and in which at the operation there was no evidence of organic disease in the colon. Both these cases appear to have been completely cured by colopexy.

*Conclusions.*—Seeing that so close a relationship exists between all the organs referred to in this paper that morbid processes taking place in any of them may affect the rest and that symptoms referred to one of them may be due to disturbance in another, the whole of these structures should, from a surgical point of view, be considered as forming part of one system. Many other parts and organs may with increasing knowledge also be found to enter into the relationship. Eventually, indeed, all the abdominal organs may come to be considered from the operative point of view as one whole. At least, as far as these particular structures are concerned, every operation should aim at a complete examination of all of them, and as far as practicable at the correction of whatever faults should be found in any of the members of the system. This will entail in every case a long incision, and the best incision is that situated to the right of the middle line with displacement of the rectus outwards. Through the wound thus made, which does no damage to the nerves or muscles of the abdominal wall, complete access can be obtained to every part of the abdominal cavity, and all these organs can be examined and dealt with without difficulty or undue disturbance to the patient.

Abnormal mobility of the proximal colon owing to the persistence of its mesentery is a factor which cannot be neglected in the surgical treatment of chronic digestive disorders, particularly in those where no gross organic disease is found at operation.

While the drag of this part of the large bowel cannot be recognised as the invariable cause of duodenal and gastric ulcers and gall-stones, it is probable that it may be one of several factors concerned in their production, and that it acts mainly through the sympathetic nervous system. The operation of colopexy should not at the present time supersede other surgical measures in the treatment of gastric and duodenal ulcers, though it may be a useful adjunct to them.

Fixation of the mobile proximal colon is worth a trial in conditions associated with mobility of the right kidney.

---

